# Smart Synthesis of Trimethyl Ethoxysilane (TMS) Functionalized Core–Shell Magnetic Nanosorbents Fe_3_O_4_@SiO_2_: Process Optimization and Application for Extraction of Pesticides

**DOI:** 10.3390/molecules25204827

**Published:** 2020-10-20

**Authors:** Khalid Al-Saad, Ahmed A. Issa, Sourour Idoudi, Basem Shomar, Mohammad A. Al-Ghouti, Nessreen Al-Hashimi, Marwa El-Azazy

**Affiliations:** 1 Department of Chemistry and Earth Sciences, College of Arts and Sciences, Qatar University, Doha 2713, Qatar; kalsaad@qu.edu.qa (K.A.-S.); ahmedissa@qu.edu.qa (A.A.I.); si1602796@student.qu.edu.qa (S.I.); nalem@qu.edu.qa (N.A.-H.); 2 Qatar Environment and Energy Research Institute (QEERI), Hamad Bin Khalifa University, Doha 2713, Qatar; bshomar@hbku.edu.qa; 3 Department of Biological and Environmental Sciences, College of Arts and Sciences, Qatar University, Doha 2713, Qatar; mohammad.alghouti@qu.edu.qa

**Keywords:** magnetic nanocomposites, trimethyl-functionalized, full factorial design (FFD), multiple responses, magnetic solid phase extraction, pesticide removal

## Abstract

In the current study, a smart approach for synthesizing trimethyl ethoxysilane–decorated magnetic-core silica-nanoparticles (TMS-mcSNPs) and its effectiveness as nanosorbents have been exploited. While the magnetite core was synthesized using the modified Mössbauer method, Stöber method was employed to coat the magnetic particles. The objective of this work is to maximize the magnetic properties and to minimize both particle size (PS) and particle size distribution (PSD). Using a full factorial design (2^k^-FFD), the influences of four factors on the coating process was assessed by optimizing the three responses (magnetic properties, PS, and PSD). These four factors were: (1) concentration of tetraethyl-orthosilicate (TEOS); (2) concentration of ammonia; (3) dose of magnetite (Fe_3_O_4_); and (4) addition mode. Magnetic properties were calculated as the attraction weight. Scanning electron microscopy (SEM) was used to determine PS, and standard deviation (±SD) was calculated to determine the PSD. Composite desirability function (D) was used to consolidate the multiple responses into a single performance characteristic. Pareto chart of standardized effects together with analysis of variance (ANOVA) at 95.0 confidence interval (CI) were used to determine statistically significant variable(s). Trimethyl ethoxysilane–functionalized mcSNPs were further applied as nanosorbents for magnetic solid phase extraction (TMS-MSPE) of organophosphorus and carbamate pesticides.

## 1. Introduction

Environmental pollution is becoming a global concern and one of the most serious apprehensions the humankind has ever faced. Seeking efficient and ecofriendly approaches to remove pollutants is becoming a challenge. Exploring the literature over the past few years, an escalating interest in nanomaterials, their synthesis and possible environmental applications can be certainly glimpsed. The focus of most of the current efforts is to have a smart synthesis of nanomaterials with high quality structures and distinctive features (e.g., magnetic, optical, catalytic, antimicrobial activities, etc.). These features would improve crucial characteristics such as permeability, hydrophilicity, selectivity, and mechanical properties, expanding the potential applications of nanomaterials in several fields [1,2,3,4].

A special attention has been paid toward magnetic nanoparticles (mNPs), where their unique magnetic features, low toxicity and hence reasonable biocompatibility, diffusibility, easy recovery, and high surface area, would open realms of applications [3,4]. Iron oxides-based mNPs (hematite α-Fe_2_O_3_, maghemite γ-Fe_2_O_3_, and magnetite Fe_3_O_4_) are among the most investigated nanosystems for wastewater treatment [5,6,7,8]. On the other hand, silica (SiO_2_) has been widely encompassed in several nanoparticles (NPs). Being non-toxic, inexpensive, easily decorated by various functional groups, and with consistent particle size (PS) and uniform particle size distribution (PSD), silica nanoparticles (SNPs) are one of the most commonly used NPs. Managing properties of SNPs such as morphology, PS, PSD, and surface charge is the key factor in controlling the different silica’s applications [9,10,11,12].

Several recent applications are based on having nanocomposites of Fe_3_O_4_@SiO_2_, i.e., a magnetic core of magnetite and a coating of silica. Having such an arrangement overcomes many difficulties associated with having either type alone, e.g., magnetite, easily degrades into maghemite within few hours of preparation. Coating of magnetite, therefore, serves to preserve both the integrity and the magnetic properties of NPs. Though SNP-based materials serve as efficient sorbents for water remediation, recovery of SNPs after the adsorption process is tiresome. Incorporation of a magnetic core, which can be removed using a magnet, would be an easier approach. Moreover, coating with silica improves features such as the liability for surface functionalization, dispersibility, and hydrophilicity [13,14].

For both types of NPs, the employed synthetic approach plays a crucial role in controlling the subsequent features and hence the applications. Several scenarios have been reported in literature for the preparation of NPs, including the sol–gel (Stöber) method [11,15,16,17,18,19,20], co-precipitation [21,22], sonolysis [23], thermal decomposition of organometallics [24], and microemulsion [25,26]. Stöber method remains the dominant choice for coating process with the following advantages: (1) good dispersibility where no surfactants exist; (2) no agglomeration compared to microemulsion-based approach; and (3) the silica coat has terminal silanol group that can bind with variety of ligands.

In the current approach, one-step procedure employing Stöber synthesis will be employed to prepare the silica coat, while the modified traditional Mössbauer method will be used for preparing the magnetic core [27]. Surveying the literature shows that the influence of the synthetic conditions on the resulting nanocomposites is controversial [11,19,20]. It can, therefore, be concluded for a one-step approach, that any uncontrolled variation would have unfavorable impacts on the process itself as well as the properties of the resulting nanocomposites.

Moreover, and since different responses would be involved (e.g., magnetic properties, PS, and PSD), it is important to consolidate these multiple responses into a single performance characteristic. Using the univariate analysis (UVA) would neither draw a comprehensive picture on the effect of variables and their interactions nor consolidate the different responses into an individual indicator. Multivariate analysis (MVA), on the contrary, can overcome these intricacies and the obtained data can be treated with a higher degree of assertion [28,29,30,31,32,33,34,35]. Therefore, the objective of this work will be to use MVA as an approach in order to assess the effect of synthetic conditions on three features of the resulting nanocomposites (responses and dependent variables: magnetic properties, PS, and PSD). Four independent variables that affect the synthetic conditions will be tested: (1) concentration of tetraethyl-orthosilicate (TEOS); (2) concentration of ammonia; (3) dose of magnetite (Fe_3_O_4_); and (4) mode of addition. A smart control of these conditions will be attained employing a two-level full factorial design (2^k^-FFD), where *k* is the number of variables to be investigated [35,36,37,38]. The output of this correlation is a mathematical paradigm that describes the impact of variations in the synthetic conditions on the measured response(s). These models also describe the magnitude and the direction of each variable’s impact.

The outcome of applying this approach is nanocomposites with high magnetic properties, controlled PS, and uniform PSD. This corollary together with features of the magnetic nanomaterials (specially the ease of separation of the adsorbent from solution using an external magnet and liability for functionalization using different moieties) compared to the conventional solid phase extraction (SPE) was further encouraging to apply the prepared nanocomposites as adsorbents for pesticides’ removal. Magnetic solid phase extraction (MSPE) as a contemporary approach surmounts the limitations of using nanomaterials, e.g., backpressure. Moreover, MSPE in this case offers better area: volume ratio, better separation dynamics, and hence higher separation capacity [39,40]. No many efforts have been seen in literature toward adjusting the synthetic conditions using factorial designs. Therefore, the novelty of this approach stems from offering a smart and green approach for optimizing the synthetic conditions of the magnetic core implementing factorial design as a platform. Nonetheless, and to the best of our knowledge, the approach used to assess the magnetic properties of the mcSNPs has not been reported before. Furthermore, nanocomposites prepared under the optimum conditions will be further decorated using trimethyl function and applied for the removal of pesticides (carbamates and organophosphorus) using gas chromatography/mass spectrometry (GC-MS). Fourier transform infrared spectroscopy (FT-IR) and thermogravimetric analysis (TGA) will be used to track the structural features of nanocomposites as well as their thermal behavior.

## 2. Results and Discussion

### 2.1. Resolution V Full Factorial Design (2^4^_V_-FFD)

Coating of previously prepared mcNPs was performed applying Stöber method [15]. A resolution V, two-level full factorial design (2^4^_V_-FFD) was instigated to tailor different scenarios of the coating process. Four variables were studied (three numerical and one categorical). The influence of these variables on three responses (PS, PSD, and magnetic properties) was investigated. Table 1 shows the variables and their upper and lower domain levels. Factorial regression was conducted for each response vs. the four variables, center points, and blocks. The impact of these variables was studied for linear (no interactions), factor–factor (quadratic), and two-way interactions. This level of resolution infers that the chosen design can estimate both main effects and factor–factor interactions without being confounded by three variable interactions (or less). The objective was set to minimize both PS (monodispersed) and PSD (evenly distributed) and maximize the magnetic properties [35]. A series of 20 experiments was created (sixteen experiments in the base block plus four added central points) in two blocks, Table 2.

### 2.2. Investigation of Variables’ Significance

**Figure 1:**
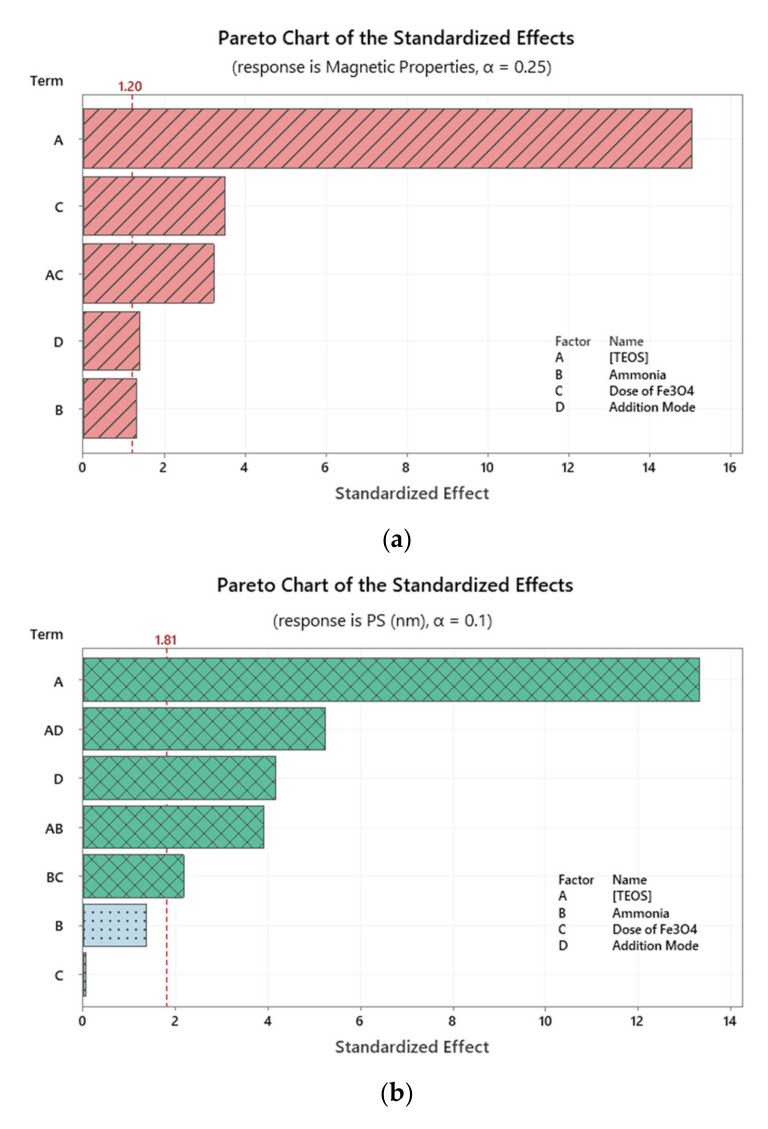

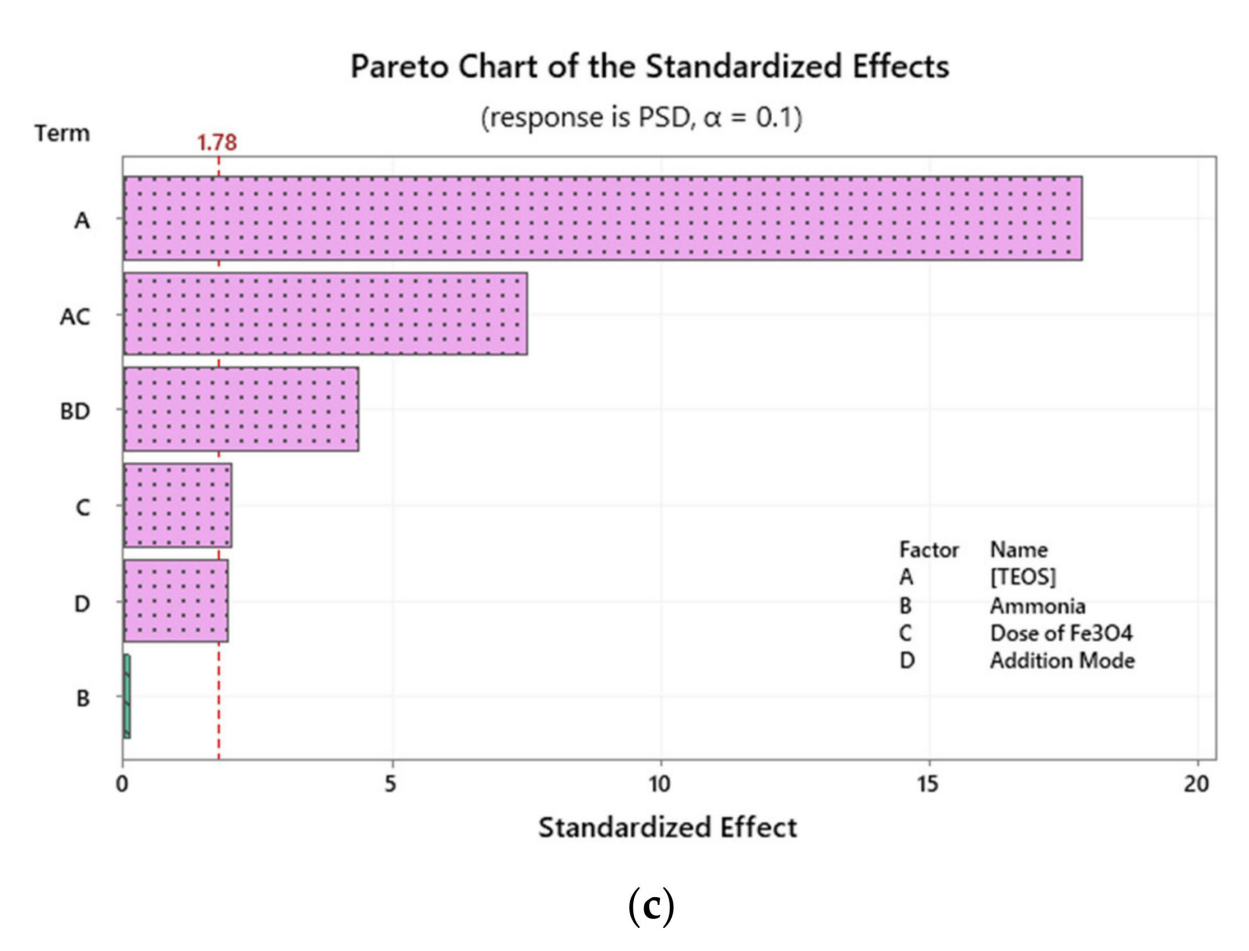
Pareto chart of standardized effects for the three measured responses. Data in each panel were obtained following response transformation. (**A**) Response is magmatic properties; (**B**) Response is PS (nm); (**C**) Response is PSD.

Pareto chart of standardized effects was used to portrait the impact of the independent variables on the three responses. As shown in Figure 1A, magnetic properties of the produced nanocomposites were mostly affected by [TEOS]. The impact of the standardized effect of [TEOS] was negative as indicated by the normal plots of standardized effects (figures are not shown). Comparably, PS and PSD were also mainly impacted by [TEOS], Figure 1B, C. Yet, the magnitude and direction of the impact of [TEOS] was different in the three responses as will be seen later in the generated regression models.

This influence of [TEOS] on the three responses could be best explained considering that while the [TEOS] increases, the amount of silica produced increases (coat thickness) and hence the PS as well as the PSD. On the other hand, this increase in the coat thickness (SiO_2_) might be masking the magnetic properties of the core. In other words, the reduction in magnetic properties might be attributed to the shielding effect input from the silica coat [4,41].

**Figure 2:**
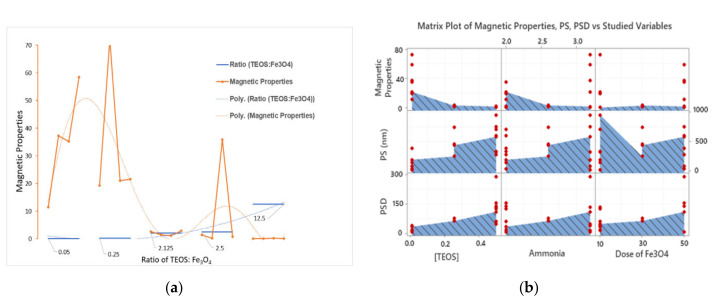
(**a**) Mapping of magnetic properties pattern as a function of the ratio of (TEOS):Fe_3_O_4_. Each of the shown ratios was obtained based on the setup shown in Table 2. Each ratio group has four points representing the four test tubes across the design setup sharing the same ratio of TEOS:Fe_3_O_4_. (**b**) A matrix plot for each of the three responses measured *vs*. the three numerical variables.

Interestingly, the influence of [TEOS] was not absolute, i.e., not every increase in the [TEOS] was associated with a decrease in magnetic properties. Three approaches were followed to verify such a pattern, namely: (1) mapping of magnetic properties vs. ratio of TEOS: Fe_3_O_4_; (2) matrix plot for each response vs. each individual variable; and (3) monitoring the magnetic properties as a response surface using two- and three-dimensional plots. Excluding the experimental errors, magnetic properties were observed to increase as the ratio of TEOS: Fe_3_O_4_ increases starting with a ratio of 1:20 and up to a ratio of 0.25:1, Figure 2A (left panel). This increase in the magnetic properties starts to diminish as the ratio moves from 2.125:1 to 2.5:1, and finally to 12.5:1. A matrix plot (every Y vs. every X) is shown on the right panel of the same graph (Figure 2B). The shown matrix reveals all possible XY combinations, where X is the independent variable(s) and Y is the measured response(s). Similar conclusions can be drawn for the impact of [TEOS]. A similar pattern can be observed for the influence of ammonia on both magnetic properties and PS.

**Figure 3:**
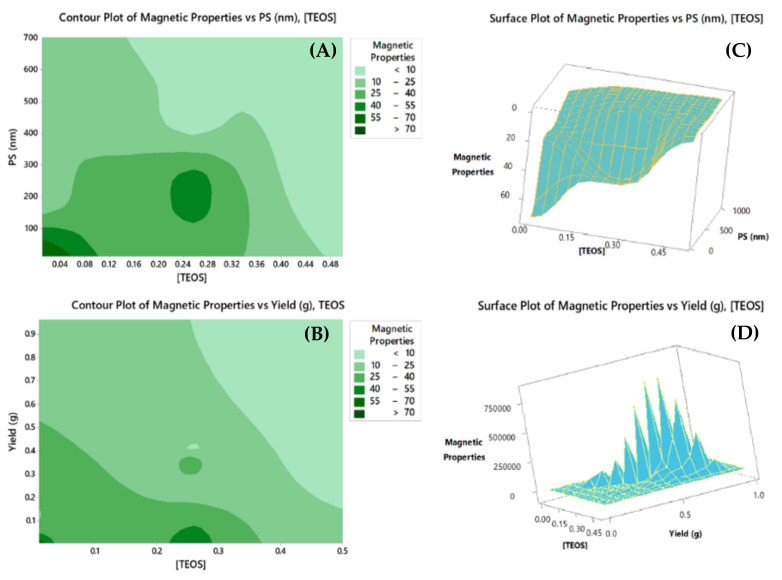
Contour and surface plots generated to study the variation in magnetic properties (z-axis) as a function of particle size (PS) (nm) and [TEOS], and yield (g) and [TEOS]. Magnetic property is considered the response variable while [TEOS], PS (nm), and yield (g) are the predictors. Figures shown are the contour plot of magnetic properties vs. (**A**) PS (nm) and [TEOS]; (**B**) Yield (g) and [TEOS] and surface plot of magnetic properties vs. (**C**) PS (nm) and [TEOS]; (**D**) Yield (g) and [TEOS].

In the same itinerary, the two-dimensional (2D) contour plots as well as surface plots (3D) were instigated to investigate the relationship between the magnetic properties on the *z-*axis (measured as response surface), and the predictors ([TEOS] and PS, upper panel; and [TEOS] and yield (g), lower panel) on the *x-* and *y-* axes, respectively, Figure 3A-D. In general and as the [TEOS] and PS (nm) increases, the magnetic property decreases (lighter areas); however, at a certain [TEOS] of nearly 0.22–0.30 M and PS of 120–280 nm, magnetic property increases again. A similar ridge was observed in the 3D surface plot. In the lower panel, a similar observation would be confirmed for both [TEOS] and yield (g).

### 2.3. Modelling and Multiple-Response Optimization

Statistical significance of studied variables was confirmed using analysis of variance (ANOVA) at 95.0 confidence interval (CI) before and following response transformation, Tables S1–S3. Similar conclusions on the statistical significance of variables were derived using ANOVA, where variables with a *p* value < 0.05 were statistically significant. The obtained regression models are shown in Equations (1)–(3) and model summaries are revealed in Table 3. It is noteworthy to mention that the three equations were obtained following response transformation. Tools such as Box–Cox transformation [42], forward selection, and backward elimination of terms were applied differently for the different responses. Transformation to normality was assessed by statistics like *p* values together with Anderson–Darling (AD) statistic and probability plots [43]. As shown in Table 3, values of R^2^ the values of R^2^ (adjusted) were relatively high, implying the model linearity. The capability of the proposed regression models to predict the responses for new observations is reflected by the high values of R^2^ (predicted).

Analysis and optimization of each response as a separate entity was a simple and a straightforward task, where each response was optimized vs. the model predictors. Yet, and with three responses being affected differently by the synthetic conditions, optimization process as a function of four variables was a tedious task. To achieve such a target, different approaches could be applied such as overlaid contour and optimization plots [38].

ln(Magnetic Properties) = 2.585 − 12.11 [TEOS] + 0.329 Ammonia + 0.0011 Dose of Fe_3_O_4_ − 0.187 Addition Mode + 0.0982 [TEOS] × Dose of Fe_3_O_4_ − 0.505 Ct Pt, (1)

ln(PS) = 6.876 − 0.89 [TEOS] − 0.874 Ammonia − 0.0281 Dose of Fe_3_O_4_+ 0.5529 Addition Mode + 1.604 [TEOS] × Ammonia − 1.286 [TEOS] × Addition Mode+ 0.01090 Ammonia × Dose of Fe_3_O_4_ + 0.634 Ct Pt, (2)

ln(PSD) = 3.445 + 1.600 [TEOS] + 0.0103 Ammonia − 0.02933 Dose of Fe_3_O_4_ − 1.023 Addition Mode + 0.0914 [TEOS] × Dose of Fe_3_O_4_ + 0.4336 Ammonia × Addition Mode + 0.549 Ct Pt, (3)

#### 2.3.1. Overlaid Contour Plots

**Figure 4:**
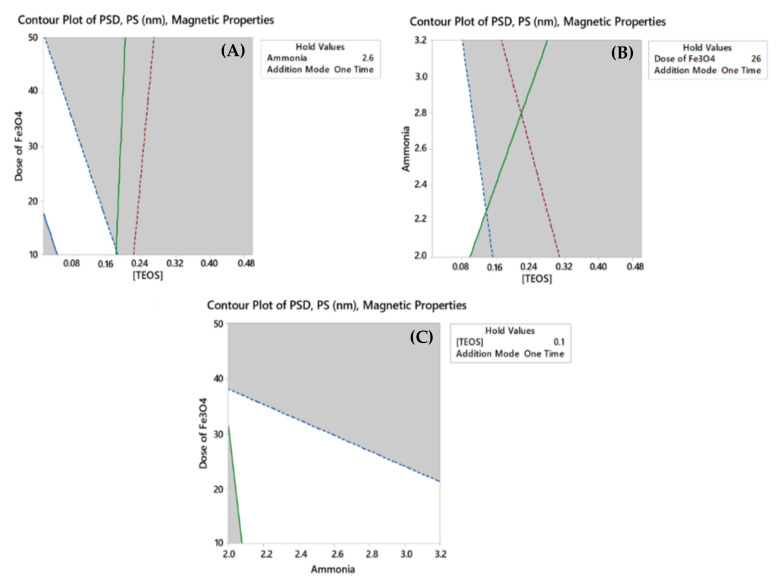
Overlaid contour plots of PSD, PS (nm) and magnetic properties for (**A**) Dose of Fe_3_O_4_ *vs* [TEOS] when ammonia concentration is held constant; (**B**) Ammonia concentration *vs* [TEOS] when dose of Fe_3_O_4_ is held constant; (**C**) Dose of Fe_3_O_4_ *vs* ammonia concentration when [TEOS] is held constant. In all cases mode of addition was held constant. The solid boundary (green, red, or blue) represent the lower limit of the response while the dashed lines are the higher frontier of the same response.

As their name implies, these types of plots are used to visually identify variables that are “viable” for multiple responses. In other words, variable settings that would satisfy (maximize, minimize, and target) one response could be different from another response. Overlaying contour plots for different responses vs. different variables could help finding a common region for each variable where the responses congregate. In the current case, the target was to maximize the magnetic properties, minimize PS, and minimize PSD; hence, lower and upper bounds were established to achieve this goal. As shown in Figure 4, contours of these boundaries vs. each pair of the numerical variables are displayed. The third numerical variable as well as the categorical variable is held at a user specific value (between −1 and +1). White areas shown on the graph represent the feasible zone for both responses [38,44]. This approach along with the consideration that different hold values would generate many graphs makes the process of visual optimization a bit tedious.

#### 2.3.2. Response Optimizer

**Figure 5:**
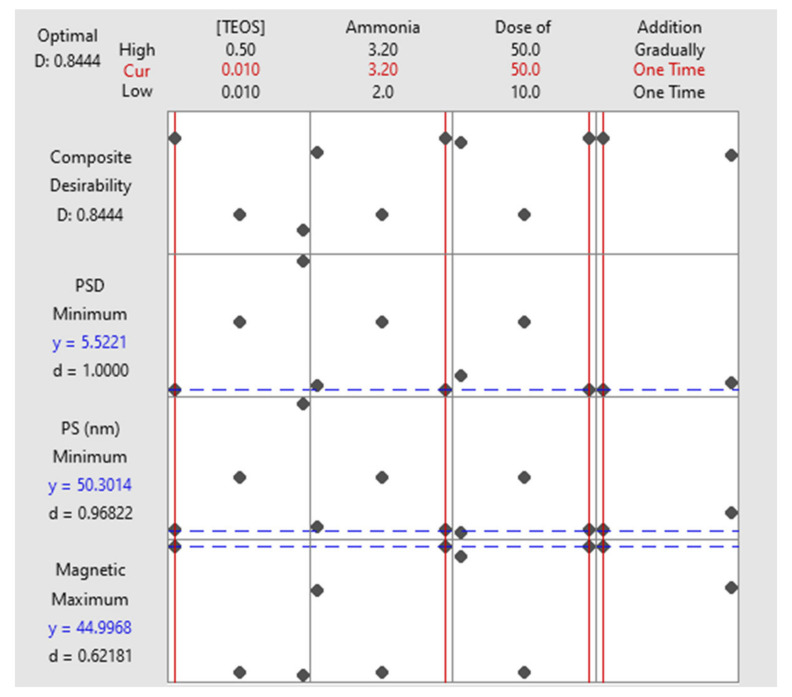
Optimization plot. The horizontal lines signify the present responses (highest magnetic properties, lowest PS, and particle size distribution (PSD)) while the upright lines are the optimal settings for each variable.

Composite desirability function (D) is an alternative approach to optimize multiple responses. Optimization plot is a graphical tool used to show how a set of experimental values affect a single response (*d*) or multiple responses (*D*). The ideal value of either *d* or D is 1.0000, and zero signifies that the shown set of variables does not represent the best arrangement for one or more responses [38,44,45]. Figure 5 is an optimization plot for the three responses where the scenario of having the highest magnetic properties with the lowest PS and PSD was the target. Obtained factorial blend of 0.01 M TEOS, 3.20 M ammonia, 50 mg/25 mL magnetite, and one-time addition mode of TEOS could achieve a PS of 50.30 nm with PSD of ± 5.52 nm and magnetic properties of ~45, with an overall desirability value of 0.8444. Applying such a blend experimentally, the actual PS was ~55.61 nm with a PSD = ± 5.98 nm (micrograph will be shown later). These findings show that there is almost no difference between the predicted and actual values.

### 2.4. Characterization of the Produced Particles

#### 2.4.1. Physical Characterization

**Figure 6:**
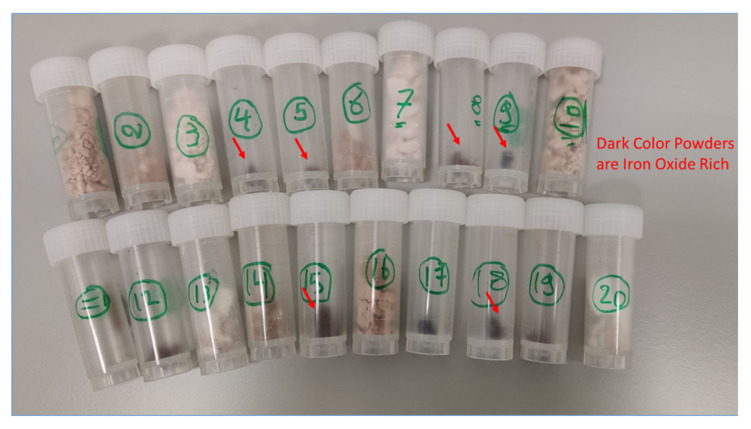
Image for the prepared adsorbents using the model described in Table 2.

Physical characterization of the prepared particles shows that the color of the produced particles ranged from white to black according to the ratio of the silica to magnetite. It was observed that as the dose of magnetite increases, the color becomes darker and vice versa as shown in Figure 6. Moreover, some of the particles were perfectly homogenous powder, while the others look like a white cone with brown powder inside. It seems like that this feature is depending on the TEOS quantity and the addition mode as shown in Table 2. The yield depends mainly on the used TEOS quantity.

#### 2.4.2. Morphology

**Figure 7:**
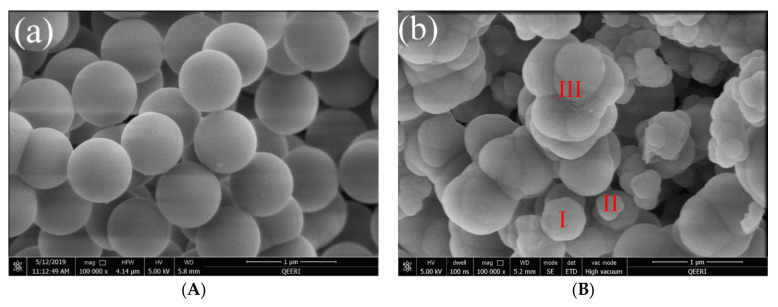
Micrographs of (**a**) well-defined and (**b**) fused particles (13 and 3, respectively, as shown in Table 2 and Figure 6).

The SEM micrographs reveal that the average particles’ diameters range from 20.85 to 947.50 nm using the experimental scenario portrayed in Table 2. Moreover, micrographs show the existence of different structures of particles varying between well-defined, fused, and growing particles, Figure 7 and Figure S1. The fused particles can be used to understand the particles’ growth, where particles with almost the same size are fused together (red II and III) to form one particle, which smooths by the ripening mechanism as (red I) [46].

#### 2.4.3. FTIR and TGA Analyses of the Produced Magnetic Nanoparticles (mNPs)

**Figure 8:**
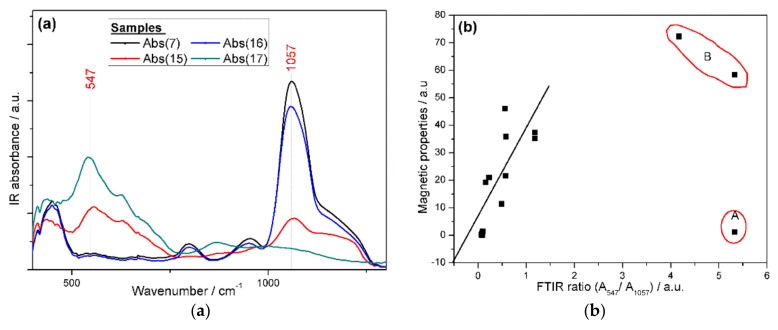
(**a**) The FTIR spectra of selected magnetic nanoparticles (mNPs) depending on its color; (**b**) relation between FTIR ratio (A_547_/ A_1057_) and magnetic properties for the prepared mNPs.

The FTIR spectra show two characteristic peaks at 547 and 1057 cm^−1^ which could be assigned to magnetite and silica, respectively. It is clear that there is a relation between the ratio of these two peaks, particles color, and, consequently, the magnetic properties of the prepared mNPs as shown in Figure 6 and Figure 8a. The relation between the ratio of the absorbances at 547 and 1057 cm^−1^ and the magnetic properties could be drawn as a straight line, Figure 8b, despite of some exceptions (marked with red on the graph), which can be attributed to experimental error. As shown in Figure 8b, for the point A, the particles are heterogenous and magnetite particles are completely separated from silica particles, while for point B, the error could be from the minute quantity of those two samples, or the relation is straight line to a certain limit.

**Figure 9:**
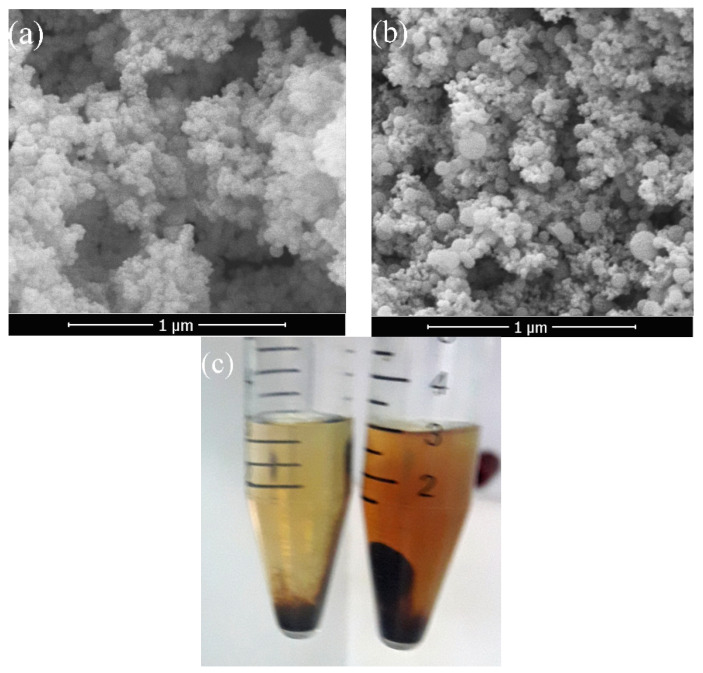
SEM micrographs for (**a**) trimethyl ethoxysilane (TMS)-mNP55 and (**b**) TMS-mNP100 while the photo in (**c**) shows the difference between magnetic decantation for both grafted mNPs (TMS-mNP55—Right and TMS-mNP100—Left).

As will be seen under the experimental section, two particles of mNPs were prepared at different conditions: optimum conditions and by setting the target PS to 100 nm, Table 4. As the size of the particles produced under the optimum condition was around 55.61 nm in diameter, these particles were suspended in the solution and were hardly collected. Therefore, the target PS was set to be above 100 nm to overcome this limitation. As expected, SEM micrographs show that PS for TMS-mNP55 was 55.60 ± 5.98 nm as revealed in Figure 9a,b. Moreover, PS has a direct influence on the magnetic decantation as shown in Figure 9c, where the small particles lead to a stable suspended solution.

**Figure 10:**
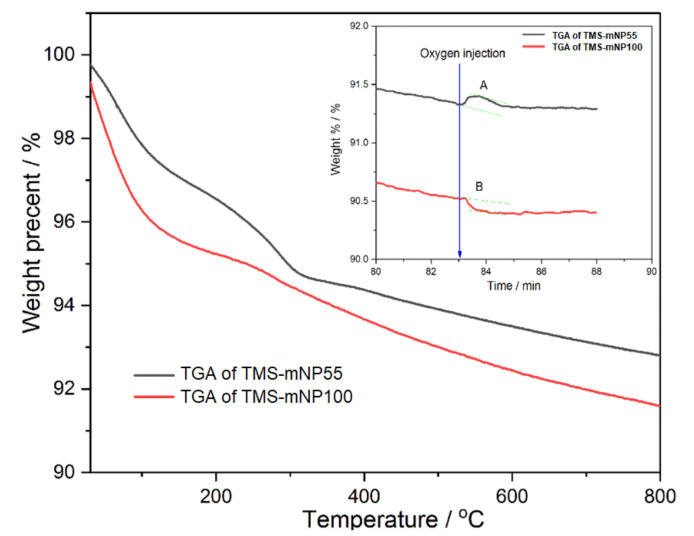
Thermogravimetric analysis (TGA) curve of A TMS-mNP55 and B TMS-mNP100.

The TGA curves of the two mNPs, Figure 10, showed different behaviors under nitrogen atmosphere, especially in the region lower than 200 °C, which revealed that the pores’ structures of the two prepared adsorbents are different. In other words, the pores in TMS-mNP100 are bigger compared to TMS-mNP55. At the introduction of oxygen gas, the weight of TMS-mNP55 increased, which could be attributed to the oxidation of magnetite to ferric oxide, while the weight of TMS-mNP100 decreased, which could be due to combustion of residual carbon as in A and B. The inset shown in Figure 10 implies that magnetite was not well shielded in TMS-mNP55.

### 2.5. Extraction of Carbamate and Organophosphorus Pesticides

#### 2.5.1. Carbamate Pesticides

**Figure 11:**
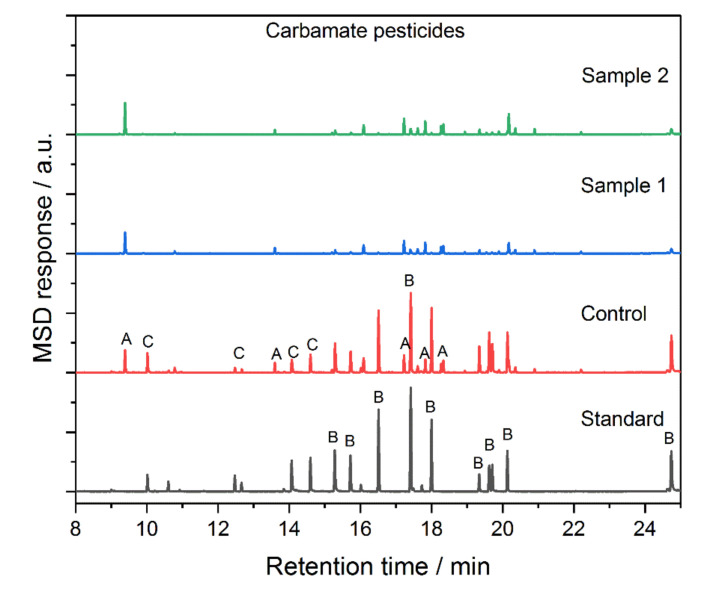
GC chromatograms of carbamate pesticides under different conditions.

The one pot–grafted mNPs (TMS-mNP55 and TMS-mNP100) were tested in the extraction of carbamate pesticides as shown in Figure 11 and Table 5. Three types of compounds can be distinguished in the chromatograms: A, B, and C. Compound (A) was formed as a result of decomposition of carbamate during the drying step, (B) represents the recoverable carbamate, while (C) is the carbamate that cannot be recovered by prepared mNPs. In general, extraction was performed better by TMS-mNP100 compared to that by TMS-mNP55. Moreover, the recovery order of the pesticides was the same in both adsorbents. It seems that the extraction of the pesticides does not follow the distribution coefficient (LogP), in contrary with what is expected. This could be attributed to other force controlling the interaction between the pesticides and grafted mcSNPs.

#### 2.5.2. Organophosphorus Pesticides

**Figure 12:**
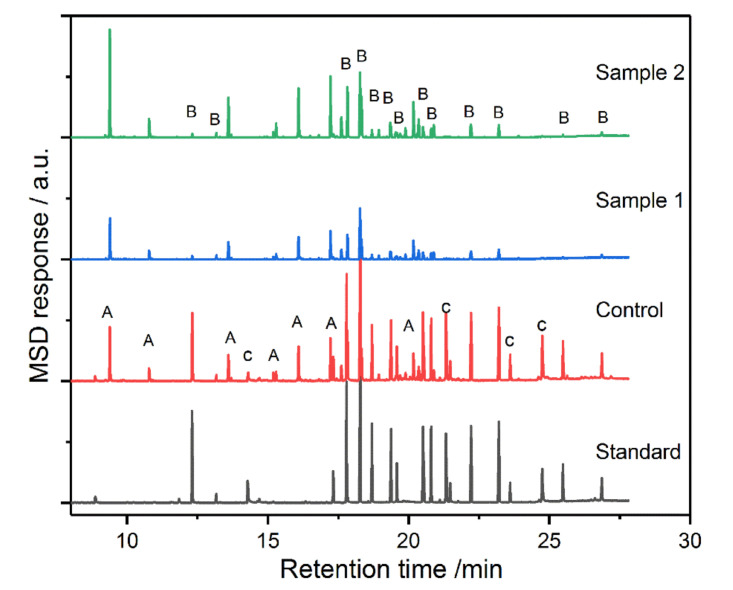
The chromatograms of organophosphorus pesticides under different conditions (sample 1: TMS-mNP55 and Sample 2: TMS-mNP100).

As in the case of carbamate pesticides, the extraction of organophosphorus pesticides by grafted mNPs in TMS-mNP100 was better than those in TMS-mNP55 (as seen in Figure 12 and Table 6). In addition, extraction did not obey the LogP. Both absorbents showed more than 10 times better extraction for dioxothion compared to the other organophosphorus pesticides.

## 3. Materials and Methods

### 3.1. Materials and Reagents

Ultrapure water (18.2 MΩ) was used for all preparations. Ammonium hydroxide with known concentration (22% *w*/*v*) was purchased from Riedel-*de*Haen^®^ Chemicals. Ammonium iron (II) sulfate hexahydrate ((NH_4_)_2_Fe(SO_4_)_2_·6H_2_O), ammonium iron(III) sulfate dodecahydrate (NH_4_Fe(SO_4_)_2_·12H_2_O), absolute ethanol (99%), dimethylformamide (DMF), sodium hydroxide (NaOH), tetraethyl orthosilicate (TEOS), and trimethyl ethoxysilane (TMS) were all analytical grade chemicals and were purchased from Sigma-Aldrich (St. Louis, MO, USA). Carbamate pesticides mixture #3 (M-CP83182A4-1 ML) and organophosphorus pesticides mixture (M-OPP16182K99-5ML) were from Chemservice GmbH (Worms, Germany).

### 3.2. Software and Instrumentation

Minitab^®^18 software (Minitab Inc., State College, PA, USA) was operated for building the implemented full factorial design (2^k^-FFD) [47]. ZEN^®^ 2.3 “Blue edition” lite Digital Imaging Software (Carl Zeiss, Promenade 10, 07745 Jena, Germany) was used to measure the diameter of the NPs in the images obtained by the scanning electron microscope (SEM). An OriginPro software (OriginLab, Northampton, MA) was utilized to execute histograms for PSD.

A Field Emission Scanning Electron Microscopy (FEI SEM, Quanta650FEG FESEM, Czech Republic: Imaging and Characterization Core Labs facility at QEERI, Hamad Bin Khalifa University, HBKU, Qatar) with a resolution power of 1.2 nm was used to describe the produced gold coated core–shell mNPs (A fine powder was dispersed onto adhesive carbon tape and sputter coated with Au.) in terms of PS, PSD, and morphology. FT-IR (Agilent, Cary 670, Santa Clara, CA, USA) with an ATR unit (PIKE, gladiATR) was used to identify the functional groups of the magnetic particles. Thermal gravimetric analyzer (TGA, PerkinElmer—TGA 400) was used to measure the decomposition of the modified NPs with respect to temperature. Analytical Balance Kern (ABT 120-5DNM) was used to determine the yield as well as the attraction weight by using neodymium magnet (96.475 g).

### 3.3. Gas Chromatography–Mass Spectrometry (GC-MS) Analysis

GC-MS (QP-2010, Shimadzu, Japan) was used to identify and measure the carbamate and organophosphorus pesticides. The separation was performed on an HP-5 MS column (30-m × 0.25 mm. i.d., 0.250 µm film thickness). The flow rate was 1.2 mL/min. Split/splitless inlet unit was used at 280 °C with split mode 10: 1. The initial oven temperature was held at 40 °C for 5 min and was ramped to 140 °C at a rate of 10 °C/min and then ramped up to 280 °C at a rate of 30 °C/min. The final temperature (280 °C) was held for 15 min. Electron impact (EI) was used as ionization source for mass spectrometry. The ion source temperature was 200 °C. The solvent delay was 8.0 min. The scan range of the MS was set at 50 to 750 *m*/*z*. The total running time for a sample ranged from 8.0 to 28.0 min.

### 3.4. Determination of Magnetic Properties

A simple setup was used to qualify the magnetic properties of the produced samples due to the unavailability of sophisticated equipment at Qatar university to measure magnetism. The setup consisted of analytical balance 5 digits (Kern & Sohn, ABT 120-5DNM, Berlin, Germany), Neodymium magnetic bar, 15 mL plastic centrifuge test tube, and a handmade copper hook. The samples’ weights were recorded as weight of yield (*w_y_*). Then the Neodymium magnet was placed on the balance that was tared, the samples were located 2 mm above the magnet in a constant position with the help of copper hook, and the reading was then recorded as weight of attraction (*w_a_*). The magnetic properties were (*w_a_*/*w_y_*) reported in Table S4 (Supporting Information). To minimize error, samples were measured in batches.

### 3.5. Procedure

#### 3.5.1. Preparation of Magnetic Core

Magnetic core NPs (mcNPs) were prepared by co-precipitation, where, 170 mL of 0.12 M aqueous solutions of both Fe (II) and Fe (III) with a molar ratio of 1:2 were added to 125 mL of 3 M NaOH solution at temperature 95 °C. The obtained black precipitate was isolated after magnetic decantation, washed with ultrapure water, acetone, and then kept in DMF. Synthesis was made in triplicate and the yield was about 50 g [27].

#### 3.5.2. Preparation of Fe_3_O_4_@SiO_2_ Nanocomposites

As previously mentioned, coating of formerly prepared mNPs were performed applying Stöber method [15]. A solution of 1 M TEOS in ethanol was freshly prepared before the coating process. The experimental scenario is shown in Table 2. One-step synthesis was performed by mixing the prerequisite amounts of ammonia, water, Fe_3_O_4_, and ethanol following the experimental setup shown in Table 2. TEOS was then added using the specified addition mode. Prepared mixtures were stirred for 1 h. The mixtures were washes three times with 10 mL DW and two times with 10 mL ethanol. Next, the mixtures were centrifuged at 4000 rpm for 30 min. Obtained precipitates were kept in oven at 70 °C for 2–3 days.

#### 3.5.3. Preparation of Functionalized Fe_3_O_4_@SiO_2_ Nanocomposites

Two adsorbents were prepared for the functionalization step as shown in Table 4. The first adsorbent (TMS-mNP55) was prepared using the optimum conditions following data analysis, while in the second adsorbent (TMS-mNP100), the target was set to adjust the PS to 100 nm. Adsorbents were then kept on the stirrer for 1 h followed by the addition of 2 mL of trimethyl ethoxysilane (TMS) to the reaction media and incubation for 1 h. on the stirrer. The grafted mNPs were collected using the magnet, washed with water and ethanol, and then dried in the oven for two days at 70 °C [48].

#### 3.5.4. Removal of Pesticides

The grafted magnetic nanoparticles (TMS-mNPs) were used to extract the carbamate (carb) and organophosphorus (org) pesticides from aqueous solutions. For this purpose, six empty centrifuge tubes were divided into two sets, one for each pesticide group. The tubes were labeled as con_carb, carb1 and carb2 for carbamates, and con_org, org1 and org2 for organophosphorus pesticides. Three portions of 1.5 mL of carb (40 ppm) pesticides were added to the carbamate tubes. Then, they were dried using nitrogen purge at 50 °C and then 1.5 mL acetone was added to the first tube as control sample. To the other two tubes, 3 mL of DI water were added as two portions, mixed well, and then 34.3 and 24.3 mg of TMS-mNP55 and TMS-mNP100 were added to carb1 and carb2, respectively. The mNPs were then collected by the magnet. The supernatant was disposed. This step was repeated one more time for washing. Finally, the collected particles were dispersed into 1.5 mL acetone for 1 min. The same steps have been conducted on the organophosphorus pesticides (100 ppm) in the second set of tubes, using 37.7 and 19.8 mg of TMS-mNP55 and TMS-mNP100 as adsorbents for org1 and org2, respectively. The mNPs were collected using the magnet and supernatants as well as the control sample were injected into the GC-MS.

## 4. Conclusions

Nanocomposites of TMS–functionalized core–shell magnetic nanoparticles (Fe_3_O_4_@SiO_2_) were successfully prepared using Massart method (magnetite core) followed by Stöber synthesis (silica coat). Optimum conditions for coating were obtained using a smart statistical approach; full factorial design (2^4^-FFD). Three responses (magnetic properties, PS, and PSD) were measured as a function of four variables. The target was to obtain minimum PS, PSD, and maximum magnetic properties. A factorial blend of 0.01 M TEOS, 3.20 M ammonia, 50 mg/25 mL magnetite, and one-time addition mode of TEOS could achieve a PS of 50.30 nm with PSD of ± 5.52 and magnetic properties of ~45, with an overall desirability value of 0.8444. Applying such a blend experimentally, the actual PS was ~55.60 nm with a PSD = ± 5.98 nm. The impact of different variables on the three responses was different. [TEOS] was the most statistically significant variable impacting the three responses. The influence of [TEOS] on the magnetic properties was not absolute and the measured response was found to be affected by the ratio of [TEOS]: dose of Fe_3_O_4_. TMS-functionalized mcSNPs were further applied as nanosorbents for MSPE of pesticides. Particles with larger PS (100 nm) were more efficient compared to smaller particles in the extraction of pesticides. This is possibly due to the formation of stable suspension, which could be attributed to their oxidation. Extraction of pesticides was not controlled by partition coefficient of pesticides.

## Figures and Tables

**Table 1:** Inspected continuous and categorical factors and their domains for a two-level (2^4^) full factorial design (FFD) instigated for the coating process.

Assessed Variables	Variable Code	Level	
		Low (−)	High (+)
Concentration of TEOS ([TEOS], M)	A	0.01	0.50
Concentration of Ammonia (Ammonia, M)	B	2.0	3.2
Dose of Fe_3_O_4_ (Dose, mg/25 mL)	C	10.0	50.0
Addition Mode	D	One time	Gradually
Responses	Highest magnetic properties Smallest PS (monodispersed) Narrowest PSD (uniform distribution)		

**Table 2:** Randomized design matrix for the 2^4^ full factorial design for coded variables. Real levels are given beside each domain code. Details of the synthetic procedure are also described.

Run#	2^4^ FFD in Coded Units with Actual Domains *					Details of the Synthetic Procedure for Each Experiment **				
	Block	A, M	B, M	C, mg/25 mL	D	Ammonia (mL)	Water (mL)	Dose of Fe_3_O_4_ (mL/25 mL)	Used TEOS (1 M)	Ethanol (mL)
01	1	0 (0.255)	0 (2.6)	0 (30)	−1 (One time)	5.4	1.04	3	6.375	9.185
02	1	+1 (0.500)	+1 (3.2)	+1 (50)	−1 (One time)	6.7	0	5	12.5	0.8
03	1	+1 (0.500)	−1 (2.0)	−1 (10)	−1 (One time)	4.2	2	1	12.5	5.3
04	1	−1 (0.010)	−1 (2.0)	+1 (50)	−1 (One time)	4.2	2	5	0.25	13.55
05	1	−1 (0.010)	−1 (2.0)	−1 (10)	+1 (Gradually)	4.2	2	1	0.25	17.55
06	1	0 (0.255)	0 (2.6)	0 (30)	+1 (Gradually)	5.4	1.04	3	6.375	9.185
07	1	+1 (0.500)	+1 (3.2)	−1 (10)	+1 (Gradually)	6.7	0	1	12.5	4.8
08	1	−1 (0.010)	+1 (3.2)	+1 (50)	+1 (Gradually)	6.7	0	5	0.25	13.05
09	1	−1 (0.010)	+1 (3.2)	−1 (10)	−1(One time)	6.7	0	1	0.25	17.05
10	1	+1 (0.500)	−1 (2.0)	+1 (50)	+1 (Gradually)	4.2	2	5	12.5	1.3
11	2	−1 (0.010)	+1 (3.2)	−1 (10)	+1 (Gradually)	6.7	0	1	0.25	17.05
12	2	+1 (0.500)	−1 (2.0)	+1 (50)	−1 (One time)	4.2	2	5	12.5	1.3
13	2	+1 (0.500)	−1 (2.0)	−1 (10)	+1 (Gradually)	4.2	2	1	12.5	5.3
14	2	0 (0.255)	0 (2.6)	0 (30)	+1 (Gradually)	5.4	1.04	3	6.375	9.185
15	2	−1 (0.010)	−1 (2.0)	+1 (50)	+1 (Gradually)	4.2	2	5	0.25	13.55
16	2	+1 (0.500)	+1 (3.2)	+1 (50)	+1 (Gradually)	6.7	0	5	12.5	0.8
17	2	−1 (0.010)	+1 (3.2)	+1 (50)	−1 (One time)	6.7	0	5	0.25	13.05
18	2	0 (0.255)	0 (2.6)	0 (30)	−1 (One time)	5.4	1.04	3	6.375	9.185
19	2	−1 (0.010)	−1 (2.0)	−1 (10)	−1 (One time)	4.2	2	1	0.25	17.55
20	2	+1 (0.500)	+1 (3.2)	−1 (10)	−1 (One time)	6.7	0	1	12.5	4.8

* A, B, C, D represent the variable’s code as defined in Table 1. ** Ethanol volume was calculated to keep the total volume constant, 25 mL. Stirring time was kept constant, 1 h for all prepared adsorbents, and addition mode (D) is the one described under the coded levels.

**Table 3:** Summaries of models revealed in Equations (1)–(3) and performed transformation.

Response	Transformation	R^2^	Adjusted R^2^	Predicted R^2^
Magnetic Properties	Box–Cox transformation (λ = 0); Forward selection of terms (α to enter = 0.25).	95.15%	92.91%	88.15%
PS	Box–Cox transformation (λ = 0); Backward elimination of terms (α to remove = 0.10)	97.08%	94.46%	88.00%
PSD	Box–Cox transformation (λ = 0); Backward elimination of terms (α to remove = 0.10)	97.22%	95.59%	91.58%

**Table 4:** Synthetic conditions for preparation of mNPs for functionalization. A volume of 250 mL of each mNPs was prepared.

Adsorbents	TEOS (mL)	Ammonia (mL)	Fe_3_O_4_ (mg/mL)	Water (mL)	Addition Mode
TMS-mNP55	0.6	66.70	50 mL (10 mg/mL)	0 mL	One time
TMS-mNP100	0.6	41.66		20 mL	One time

**Table 5:** The extracted concentrations of carbamate pesticides using TMS-mNP55 and TMS-mNP100 from 1.5 mL of the contaminated samples.

Carbamate Pesticides (Stock Solution 40 ppm)	R_t_ (min.)	Control		Extracted (ppm)	
		ppm	%	TMS-mNP55	TMS-mNP100
Aldicarb	9.091	35.90	89.75	0	0
Dioxacarb	14.08	19.77	49.42	0.182	0.263
Methiocarb sulfoxide	15.73	28.98	72.44	2.406	2.651
Propoxur	16.52	34.80	86.99	0.604	0.899
Promecarb	17.42	34.82	87.06	1.706	2.587
Carbofuran	18.01	42.27	105.7	0.631	1.165
Dioxacarb	19.35	73.26	183.2	8.948	13.52
3-Hydroxycarbofuran	19.63	73.94	184.8	0.181	0.296
Methiocarb	20.14	46.78	116.9	12.28	23.61

**Table 6:** The extracted concentrations of the organophosphorus pesticides by using TMS grafted mNPs: TMS-mNP55 and TMS-mNP100 from 1.5 mL contaminated samples.

R_t_ (min.)	Organophosphorus Pesticide	Stock	Control	%	TMS-mNP55	TMS-mNP100
		Conc. (ppm)	Conc. (ppm)		ppm	ppm
**14.29**	**Mevinphos**	10	4.065	40.65	0	0
17.32	Sulfotep	5	3.955	79.10	0.294	0.468
17.80	Demeton-S	20	16.96	84.79	0.072	0.152
18.28	Dioxothion	60	52.24	87.06	15.67	15.90
18.70	Disulfoton	10	7.095	70.95	0.535	0.875
19.37	Dichlofenthion	10	7.484	74.84	0.851	1.276
20.51	Fenthion	10	8.974	89.74	0.830	1.278
20.80	Trichloronat	10	7.739	77.39	0.857	1.153
21.10	Clofenvinfos	5	5.318	106.4	1.520	1.851
21.33	Crotoxyphos	20	20.99	105.0	0	0.027
22.22	Prothiofos	10	9.641	96.41	0.796	1.174
23.20	Ethion	10	9.623	96.23	1.129	1.442
23.61	Famphur	20	25.98	129.9	0	0
24.75	Phosmet	20	21.29	106.5	0	0
25.48	Leptophos	10	10.58	105.8	0.254	0.435

## Supplementary Materials

The following are available online at https://www.mdpi.com/1420-3049/25/20/4827/s1: Table S1: Analysis of variance for transformed response in case measured response is magnetic properties. Table S2: Analysis of variance for transformed response in case measured response is PS. Table S3: Analysis of variance for transformed response in case measured response is PSD. Table S4: Physical properties of the produced samples. Figure S1: Micrographs of prepared samples as in Table S4.
