# Supplementary material for: Smart Synthesis of Trimethyl Ethoxysilane (TMS) Functionalized Core–Shell Magnetic Nanosorbents Fe3O4@SiO2: Process Optimization and Application for Extraction of Pesticides
Source: Molecules. 2020 Oct 20;25(20):4827. doi: 10.3390/molecules25204827 (PMC7587953; doi:10.3390/molecules25204827)
Supplement: Supplementary file 1 [file molecules-25-04827-s001.pdf]

Supporting information

Table S1. Analysis of Variance for Transformed Response in Case Measured Response is Magnetic Properties.

Source	DF	Adj SS	Adj MS	F-Value	P-Value
**Model**	6	90.9068	15.1511	42.50	0.000
**Linear**	4	86.3826	21.5957	60.57	0.000
**[TEOS]**	1	80.6953	80.6953	226.33	0.000
**Ammonia**	1	0.6240	0.6240	1.75	0.209
**Dose of Fe3O4**	1	4.3607	4.3607	12.23	0.004
**Addition Mode**	1	0.7026	0.7026	1.97	0.184
**2-Way Interactions**	1	3.7080	3.7080	10.40	0.007
**[TEOS]*Dose of Fe3O4**	1	3.7080	3.7080	10.40	0.007
**Curvature**	1	0.8162	0.8162	2.29	0.154
**Error**	13	4.6349	0.3565		
**Total**	19	95.5417			

Table S2. Analysis of Variance for Transformed Response in Case Measured Response is PS.

Source	DF	Adj SS	Adj MS	F-Value	P-Value
**Model**	9	19.3435	2.1493	36.97	0.000
**Blocks**	1	3.8536	3.8536	66.29	0.000
**Linear**	4	11.4534	2.8633	49.26	0.000
**[TEOS]**	1	10.3314	10.3314	177.72	0.000
**Ammonia**	1	0.1099	0.1099	1.89	0.199
**Dose of Fe3O4**	1	0.0002	0.0002	0.00	0.951
**Addition Mode**	1	1.0118	1.0118	17.41	0.002
**2-Way Interactions**	3	2.7518	0.9173	15.78	0.000
**[TEOS]*Ammonia**	1	0.8894	0.8894	15.30	0.003
**[TEOS]*Addition Mode**	1	1.5887	1.5887	27.33	0.000
**Ammonia*Dose of Fe3O4**	1	0.2736	0.2736	4.71	0.055
**Curvature**	1	1.2848	1.2848	22.10	0.001
**Error**	10	0.5813	0.0581		
**Total**	19	19.9248			

Table S3. Analysis of Variance for Transformed Response in Case Measured Response is PSD.

Source	DF	Adj SS	Adj MS	F-Value	P-Value
**Model**	7	23.8228	3.4033	59.87	0.000
**Linear**	4	18.5640	4.6410	81.64	0.000
**[TEOS]**	1	18.1160	18.1160	318.69	0.000
**Ammonia**	1	0.0006	0.0006	0.01	0.919
**Dose of Fe3O4**	1	0.2315	0.2315	4.07	0.066
**Addition Mode**	1	0.2159	0.2159	3.80	0.075
**2-Way Interactions**	2	4.2952	2.1476	37.78	0.000
**[TEOS]*Dose of Fe3O4**	1	3.2124	3.2124	56.51	0.000
**Ammonia*Addition Mode**	1	1.0827	1.0827	19.05	0.001
**Curvature**	1	0.9636	0.9636	16.95	0.001
**Error**	12	0.6821	0.0568		
**Total**	19	24.5049			

Table S4. Physical properties of the produced samples

# ID	Yield (Wy)	Attraction weight (Wa)	Ratio (Wa/ Wy)	FTIR ratio	Notes		
	gm	gm			color/state	Homogeneity	volume
1	0.72152	1.8781	2.6030	0.072716136	brown powder (+)	Homogenous	around 5 ml
2	0.3569	0.528	1.479405996	0.088856607	brown powder (++)	there are pieces of white particles (silica)	around 2.5 ml
3	0.7215	0.074	0.102564103	0.072716136	off white powder	Homogenous	around 5 ml

4	0.0978	1.1194	11.44580777	0.487067362	Brown powder (+++)	homogenous	0.5 ml
5	0.00991	0.1914	19.31382442	0.154508354	brown powder (+++)	homogenous	0.1 ml
6	0.36452	0.5142	1.410622188	0.103458844	white cone with internal brown color (+++)	composed of two separate homogenous regions	2 ml
7	0.698	0.0897	0.128510029	0.084808348	off white powder	Homogenous	5 ml
8	0.03671	1.3695	37.3059112	1.177379076	Brown powder (++++)	Homogenous	0.3 ml
9	0.00414	0.29938	72.31400966	4.166648051	almost black powder	Homogenous	0.05 ml
10	0.96211	0.2643	0.274708713	0.091625115	brown powder	Homogenous	6 ml
11	0.00958	0.2017	21.05427975	0.230286364	Brown (+++) powder	Homogenous, as there is electrostatic charge on the particles	0.1 ml
12	0.034	1.2202	35.88823529	0.581771044	Brown powder (++-)	Homogenous	0.2 ml
13	0.73767	0.143	0.19385362	0.067054283	solid white cone with internal brown color (+++)	composed of two separate homogenous regions	2.5 ml
14	0.3653	0.4153	1.136873802	5.334406727	brown (+++) powder with white pieces	heterogeneous	1.0 ml

15	0.03749	1.3197	35.20138704	1.177379076	Brown powder (+++-)	Homogenous	0.2 ml
16	0.71152	0.578	0.812345401	0.084405797	Brown (++) powder	Homogenous	4.0 ml
17	0.03368	1.9699	58.48871734	5.334406727	almost black powder	Homogenous	0.2 ml
18	0.01661	0.04949	2.9800	0.557276853	Brown (++++) powder	Homogenous (particles as have electrostatic charges)	0.2 ml
19	0.00858	0.1853	21.5967366	0.570663012	Brown(+++) powder	Homogenous	0.2 ml
20	0.91679	0.0763	0.083225166	0.088856607	white cone with internal brown color (++)	composed of two separate homogenous regions	3 ml

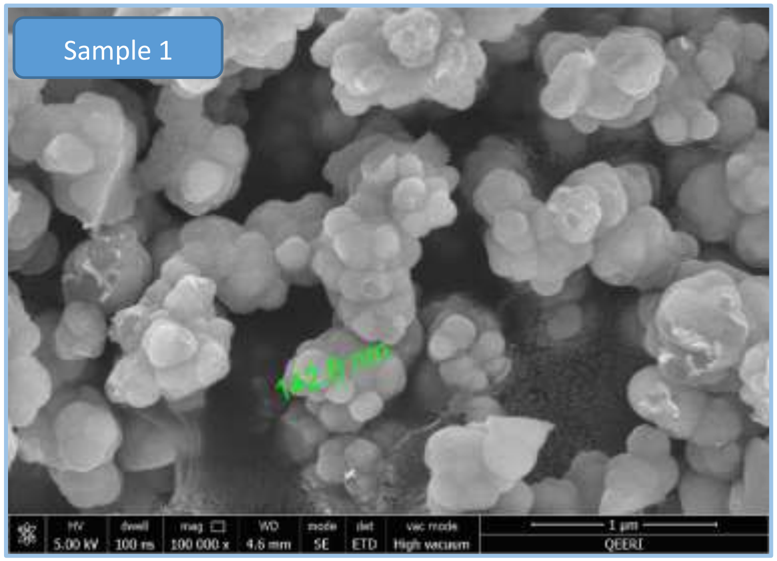

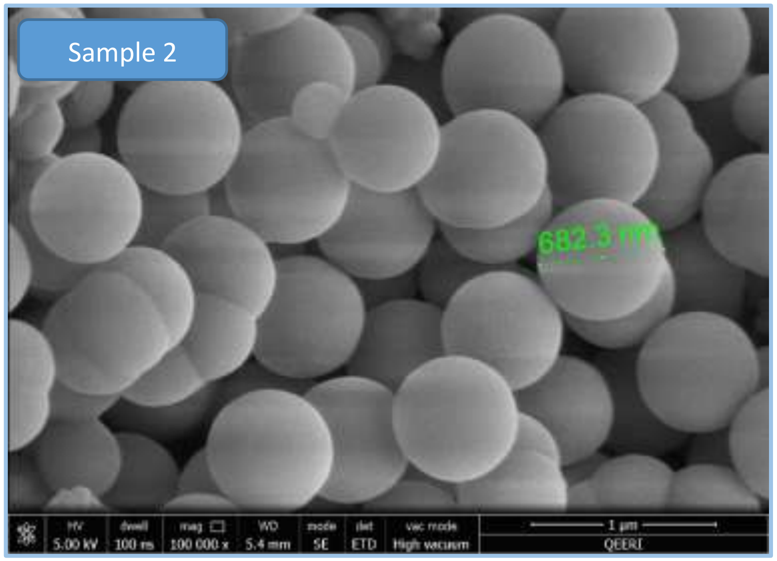

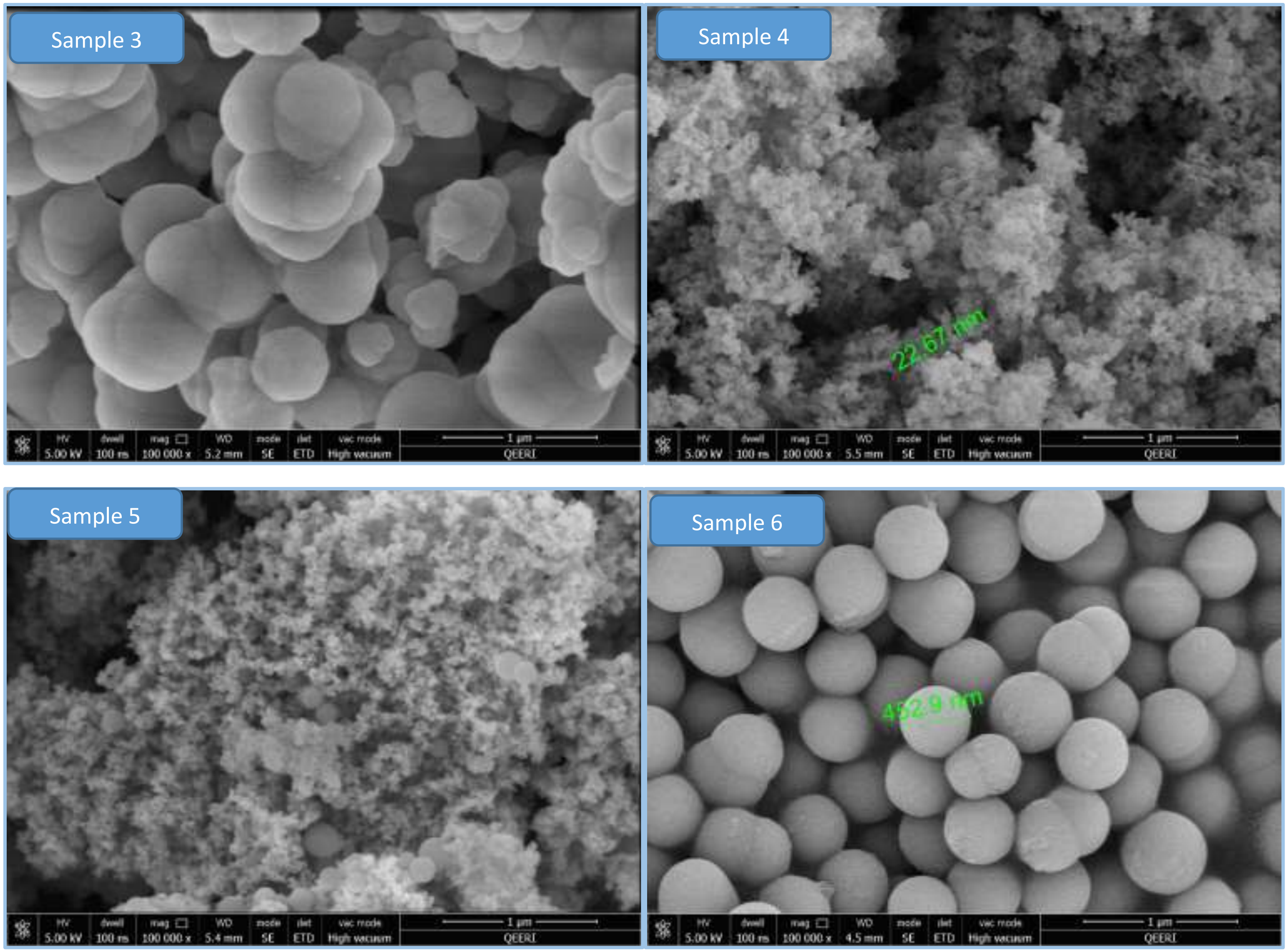
Sample 3
HV 5.00 kV dwell 100 ns mag 100 000 x WD 5.2 mm mode SE det ETD vac mode High vacuum
1 µm
QEERI
Sample 4
22.67 nm
HV 5.00 kV dwell 100 ns mag 100 000 x WD 5.5 mm mode SE det ETD vac mode High vacuum
1 µm
QEERI
Sample 5
HV 5.00 kV dwell 100 ns mag 100 000 x WD 5.4 mm mode SE det ETD vac mode High vacuum
1 µm
QEERI
Sample 6
452.9 nm
HV 5.00 kV dwell 100 ns mag 100 000 x WD 4.5 mm mode SE det ETD vac mode High vacuum
1 µm
QEERI

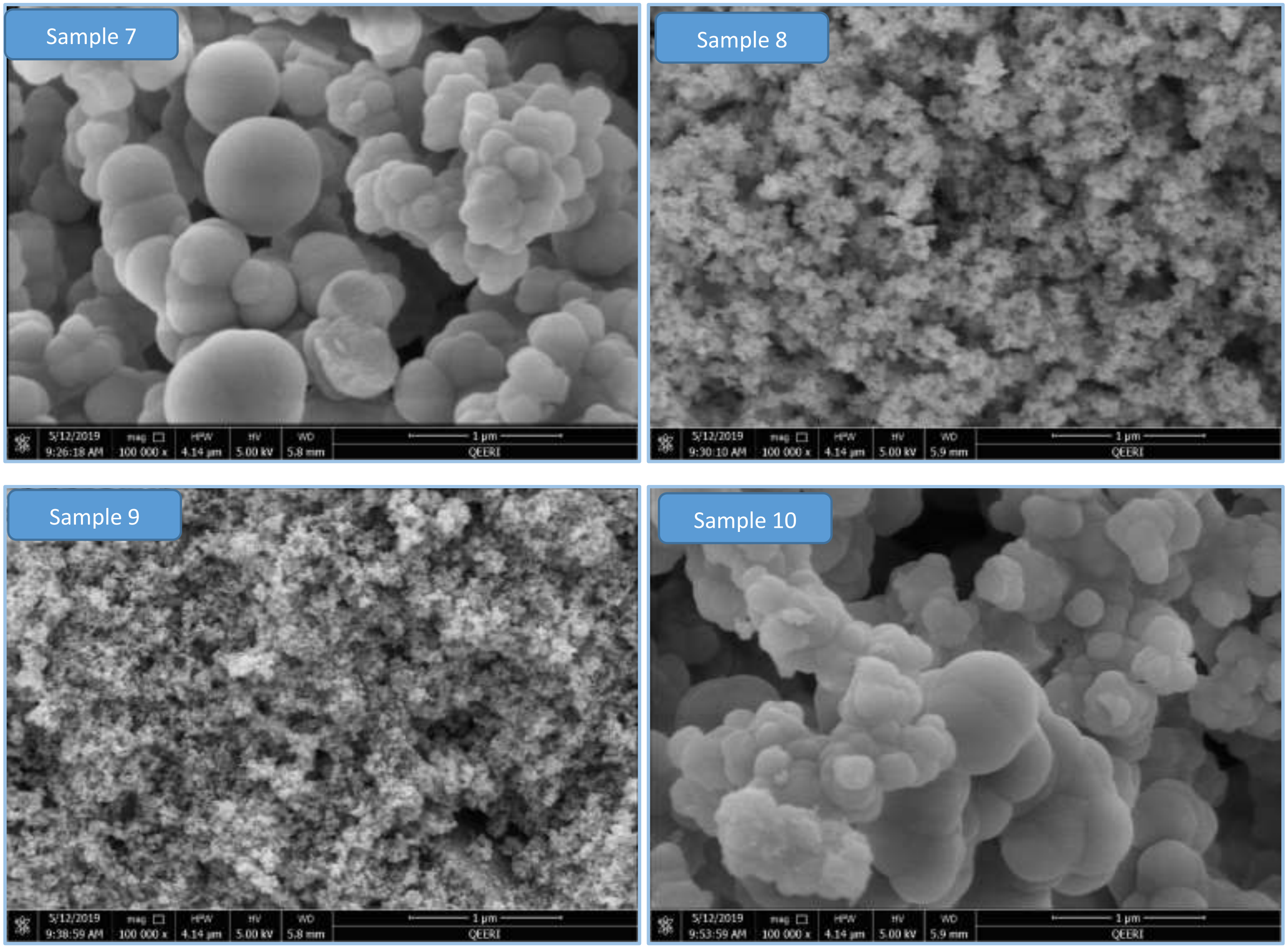
Sample 7
5/12/2019 9:26:18 AM
mag 100 000 x
HFW 4.14 µm
HV 5.00 kV
WD 5.8 mm
1 µm
QEERI
Sample 8
5/12/2019 9:30:10 AM
mag 100 000 x
HFW 4.14 µm
HV 5.00 kV
WD 5.9 mm
1 µm
QEERI
Sample 9
5/12/2019 9:38:59 AM
mag 100 000 x
HFW 4.14 µm
HV 5.00 kV
WD 5.8 mm
1 µm
QEERI
Sample 10
5/12/2019 9:53:59 AM
mag 100 000 x
HFW 4.14 µm
HV 5.00 kV
WD 5.9 mm
1 µm
QEERI

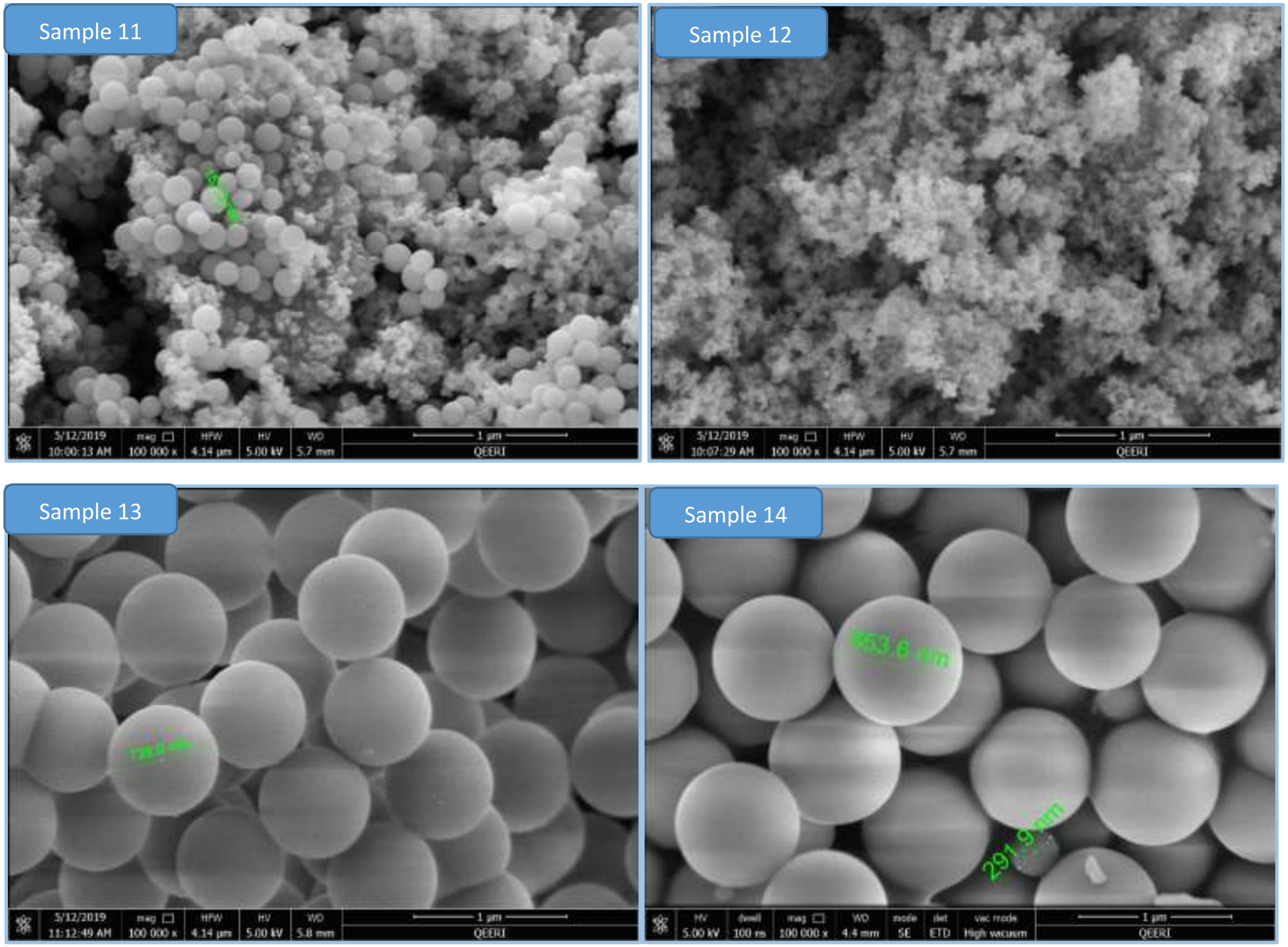
Sample 11
5/12/2019 10:00:13 AM mag 100 000 x HFW 4.14 µm HV 5.00 kV WD 5.7 mm 1 µm QEERI
Sample 12
5/12/2019 10:07:29 AM mag 100 000 x HFW 4.14 µm HV 5.00 kV WD 5.7 mm 1 µm QEERI
Sample 13
5/12/2019 11:12:49 AM mag 100 000 x HFW 4.14 µm HV 5.00 kV WD 5.8 mm 1 µm QEERI
Sample 14
853.6 nm
291.9 nm
HV 5.00 kV dwell 100 ns mag 100 000 x WD 4.4 mm mode SE det ETD vac mode High vacuum 1 µm QEERI

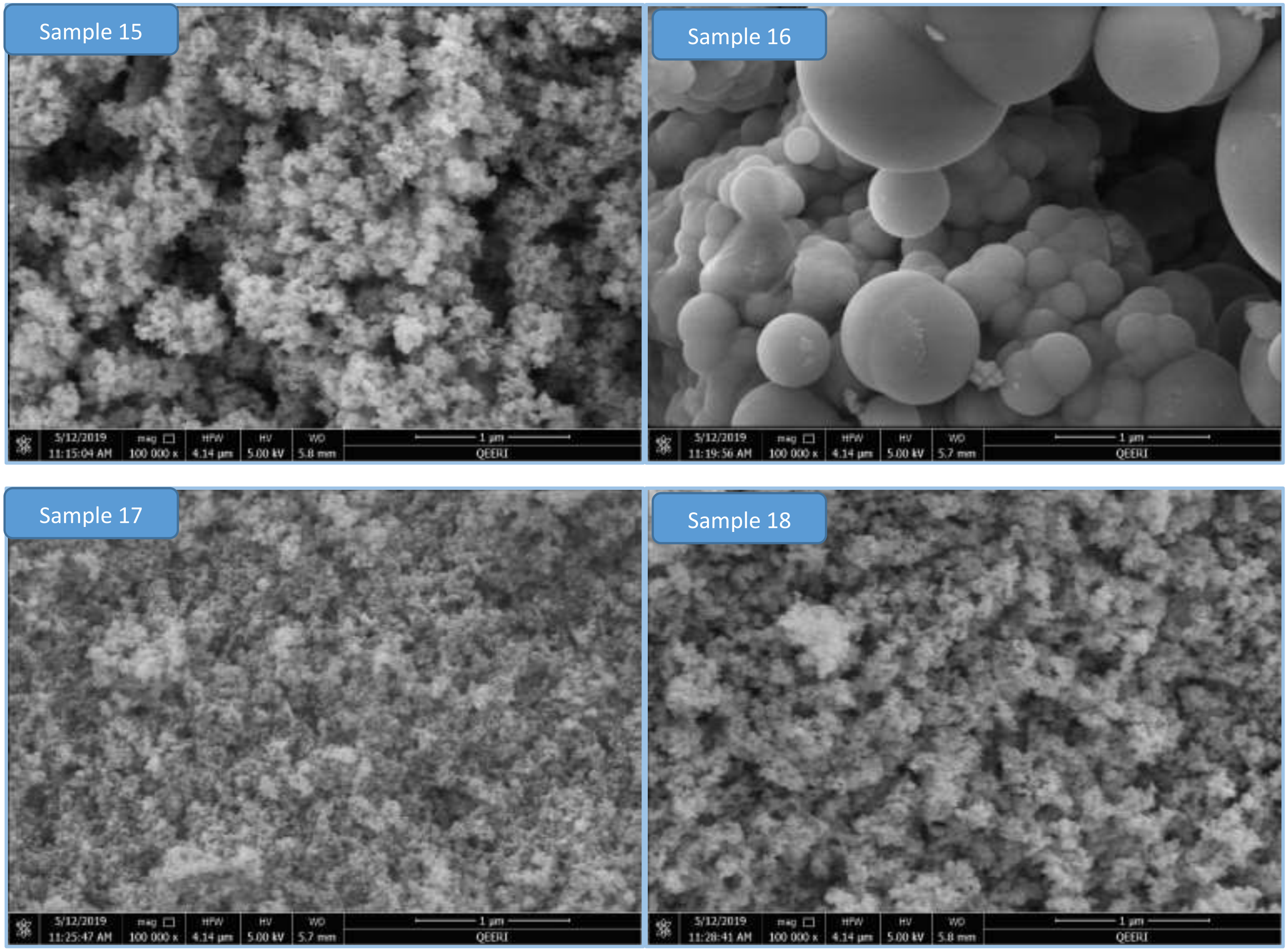
Sample 15
5/12/2019 11:15:04 AM | mag 100 000 x | HFW 4.14 µm | HV 5.00 kV | WD 5.8 mm | 1 µm | QEERI
Sample 16
5/12/2019 11:19:56 AM | mag 100 000 x | HFW 4.14 µm | HV 5.00 kV | WD 5.7 mm | 1 µm | QEERI
Sample 17
5/12/2019 11:25:47 AM | mag 100 000 x | HFW 4.14 µm | HV 5.00 kV | WD 5.7 mm | 1 µm | QEERI
Sample 18
5/12/2019 11:28:41 AM | mag 100 000 x | HFW 4.14 µm | HV 5.00 kV | WD 5.8 mm | 1 µm | QEERI

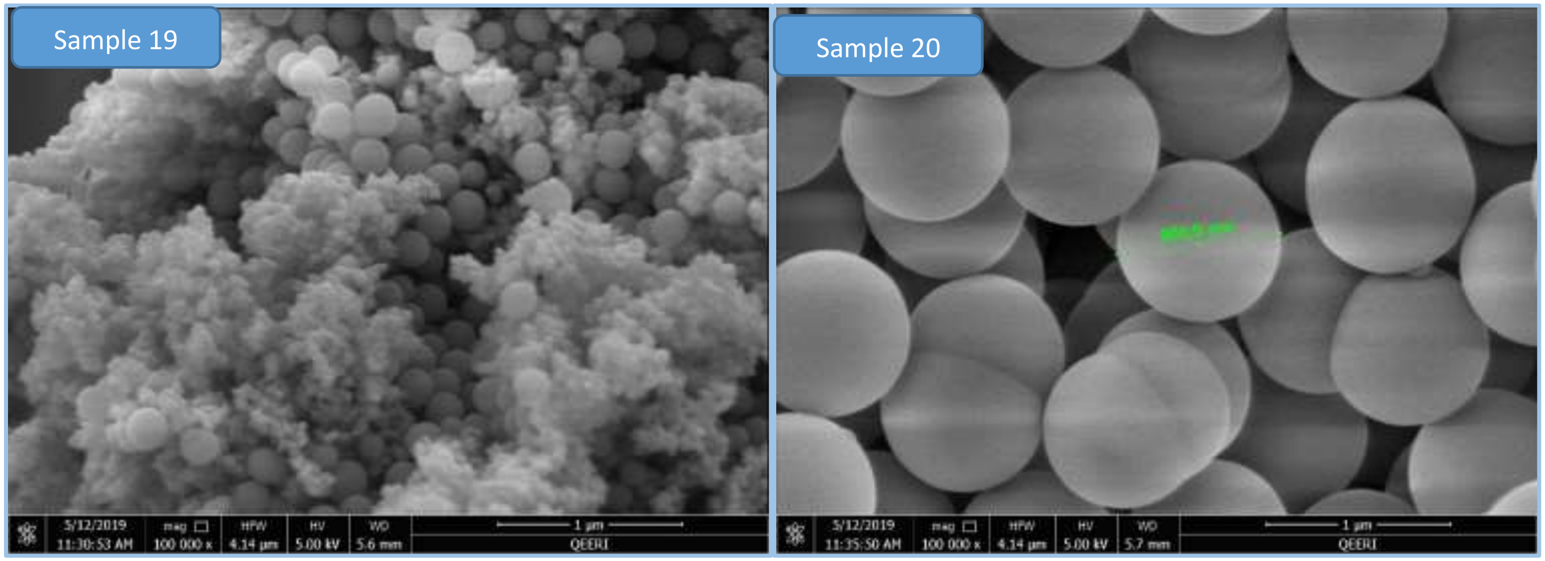

Figure S1. Micrographs of prepared samples as in Table S4.
